# Evaluation of Online Palliative Care Training for Certified Nursing Assistants

**DOI:** 10.1093/geroni/igaa057.1680

**Published:** 2020-12-16

**Authors:** Jinsook Kim, Jennifer Gray

**Affiliations:** Northern Illinois University, DeKalb, Illinois, United States

## Abstract

Palliative care for older adults is increasingly needed due to a burgeoning older adult population. Certified nursing assistants (CNAs) in skilled nursing facilities (SNFs) provide assistance with activities of daily living and comfort care. There, however, is a significant gap in evaluated palliative care trainings for CNAs. We used a waitlisted control group design to evaluate the effectiveness of an 8-module online palliative care training. CNAs (n=102) from 6 SNFs were randomly assigned to an experimental (n=51) and a control group (n=51) and completed a baseline evaluation. The experimental group took a posttest about palliative care knowledge upon training completion and a 1-month follow-up assessment about palliative care self-efficacy. The control group completed the assessments at the same time as the experimental group prior to receiving the training. The majority of the participants were female (92%). On average, participants were 31 years old, with 6.5 years tenure in the field. The retention rate was 90% at the posttest (n=92) and 82% at the 1-month follow-up (n=84). Palliative care knowledge (scored 0–100) significantly increased in the experimental group (mean 4.1, p < 05), with no significant change in the control group. Palliative care self-efficacy (scored 20-100) significantly improved from the baseline to follow-up in both groups (mean 4.3 and 5.8 respectively, p < 05) with no significant difference between study groups. The results indicate the effectiveness of an online palliative care training to improve CNA knowledge. Improvement in palliative care self-efficacy regardless of training participation warrants further exploration.

